# The Role of Video Capsule Endoscopy in Hereditary Polyposis Syndromes: A Narrative Review

**DOI:** 10.3390/diagnostics15212813

**Published:** 2025-11-06

**Authors:** Magdalini Manti, Faidon-Marios Laskaratos, Andrew Latchford, Kevin Monahan, Owen Epstein, Adam Humphries

**Affiliations:** 1 St Mark’s National Bowel Hospital, Acton Ln, London NW10 7NS, UK; flaskaratos@nhs.net (F.-M.L.); andrew.latchford@nhs.net (A.L.); k.monahan@nhs.net (K.M.); adam.humphries@nhs.net (A.H.); 2 Department of Surgery and Cancer, Faculty of Medicine, Imperial College London, London W12 0NN, UK; 3 The Polyposis Registry, St Mark’s Centre for Familial Intestinal Cancer, St Mark’s National Bowel Hospital, Acton Ln, London NW10 7NS, UK; 4 Royal Free Hospital, Pond St., London NW3 2QG, UK; owen@eppifam.co.uk

**Keywords:** capsule endoscopy, polyposis syndrome, PJS, FAP

## Abstract

Video Capsule Endoscopycapsule endoscopy (VCE) has emerged as a minimally invasive diagnostic tool for detecting and monitoring small bowel involvement in polyposis syndromes. VCE is included in the surveillance guidelines of Peutz-Jeghers syndrome. In the remaining familial polyposis syndromes, VCE may facilitate the early detection of polyps, when indicated, particularly in areas beyond the reach of conventional endoscopy, thereby aiding timely detection. Colon capsule endoscopy has been studied in symptomatic, screening and polyp surveillance populations and the second-generation colon capsule has demonstrated excellent detection rates for advanced neoplasia, however its role in colonic polyposis requires further research. The role of the panenteric capsule has not been explored in polyposis syndromes as a panintestinal examination. Despite its advantages, VCE has notable limitations; it may miss small, flat, or hidden lesions and lacks the capability for tissue sampling or therapeutic intervention. In the future, advances in imaging technology, extended battery life, and the integration of artificial intelligence (AI) are expected to further enhance the utility of VCE. Our review aims to focus on the applications of VCE in polyposis syndromes and future perspectives.

## 1. Introduction

Video capsule endoscopy (VCE) was introduced at the beginning of the 21st century following its conception and development by Gavriel Iddan [1]. The main purpose of small bowel capsule endoscopy is to visualise the small bowel after the second part of the duodenum, but more recently capsule endoscopes have been developed specifically for the assessment of the upper gastrointestinal tract, colon and panenteric examination [2]. Capsule endoscopy is a minimally invasive test that involves the patient swallowing a small capsule equipped with a camera. In most capsule endoscopy systems, an external recorder captures the images transmitted by the capsule as it travels through the digestive tract. Since its initial introduction in 2001, various systems for capsule endoscopy have been developed and released to the market.

Several small bowel capsule endoscopy (SBCE) systems are commercially available and include the PillCam *SB3* [3] (Medronic, Dublin, Ireland), the MiroCam 4000 [4] (Intromedic, Seoul, Korea), the Endocapsule 10 [2] (Olympus, Tokyo, Japan), the Capsocam Plus [5] (Capsovision, Saratoga, NY, USA) [2] and the OMOM HD [6] (Jinshan science & technology, Chongqing, China). A list of the small bowel capsule endoscopy systems which are currently or have previously been commercially available is summarised in Table 1.

The first generation, PillCam COLON^®^ (Given Imaging, Medtronic, Dublin, Ireland), was released in the market in 2006 [21,22]. In 2009, PillCam COLON2^®^ (Given Imaging, Medtronic, Dublin, Ireland) was released capturing up to 35 frames per second, while in 2017 the Panenteric PillCam Crohn’s^®^ (Given Imaging, Medtronic, Dublin, Ireland) was marketed with a 344°-wide view of the mucosa and a prolonged battery capacity of over 12 h, which allows assessment of both the small and large bowel [21,23].

The use of colon capsule endoscopy in polyposis syndromes requires further research. Colon capsule endoscopy has been employed in the large bowel for polyp detection and bowel cancer screening, after incomplete or unfeasible ileo-colonoscopy and this clinical use also aligns with the ESGE guidelines [24]. Colon capsule endoscopy avoids some complications associated with standard colonoscopy, such as perforation and sedation risks [2]; however, it is purely a diagnostic tool, as additional bowel preparation is needed and completion rates vary between 68 and 98%. It is considered a third-line investigation in colorectal cancer screening by the American Multi-Society Task Force, after annual faecal immunochemical test (FIT) with 10-yearly colonoscopy (first line) and 5-yearly CT-colonography with 3-yearly FIT-faecal DNA test (second line) [25].

The objective of this review was to summarise the current application of video capsule endoscopy in the management of hereditary polyposis syndromes.

## 2. Materials and Methods

We performed a narrative, non-systematic literature review synthesizing published evidence on VCE in hereditary polyposis syndromes. The literature search included online sources from Pubmed and Scopus. The search was performed from March 2024 until June 2025 and included the terms “capsule endoscopy”, “colon capsule” as medical subject heading (MeSH) and free-text terms. These results were combined using the Boolean set operator “AND” with the term “polyposis syndromes” as a MeSH and free-text term. The initial electronic search was followed by a manual search of references from retrieved studies to identify additional suitable bibliography.

Regarding the inclusion criteria, human studies and guidelines/position statements addressing VCE (small-bowel, colon, or panenteric) in hereditary polyposis syndromes were used in our narrative review. Study designs included randomised and non-randomised studies, retrospective cohort studies, case series/reports, and reviews/guidelines providing syndrome-specific data.

We excluded articles not written in English, editorials/personal opinions without primary data, studies without accessible full text, animal studies, and articles focusing on VCE for non-polyposis indications. Articles excluded as “irrelevant” met one or more of the above exclusion reasons.

186 results were found and analysed. After removing duplicate records (*n* = 8), 178 articles were initially screened. Five articles were written in non-English language (Korean, Portuguese, Czech, Hungarian, and Spanish), two articles were not retrieved, one article compared VCE among inpatients and outpatients, and 70 articles were irrelevant. Finally, 100 articles were included in the study.

The SBCE generation classification was based on the characteristics of the capsules, as per the manufacturer, and the date of the capsule release in the market. First-generation capsules (*SB1*) (i.e., OMOM capsule [13], Capsocam SV-1 [14], EndoCapsule [10], PillCam SB [7,8,9,10]) featured a single camera with a narrow field of view (140°), fixed frame rate (2 frames per second, FPS), and limited battery life, providing basic but revolutionary small-bowel visualisation [26]. Second-generation systems (*SB2*, including PillCam *SB2* [11,12] and EndoCapsule 10 [2]) introduced enhanced optics, adaptive illumination, wider viewing angles (up to 170°), improved image resolution and transmission stability [26,27]. Third-generation capsules (*SB3*, e.g., PillCam *SB3* [3]) further optimised frame rate (2–6 FPS adaptive), increased sensitivity and dynamic brightness control, and allowed improved localisation and review software, resulting in higher lesion detection rates [28].

## 3. Inherited Polyposis Syndromes

Polyposis syndromes can occur as hereditary conditions or may arise sporadically. In cases where the small intestine is affected, the use of SBCE is at its most valuable.

Below we describe the use of VCE in hereditary polyposis syndromes [29] (Table 2).

### 3.1. Peutz-Jeghers Syndrome (PJS)

**Figure 1:**
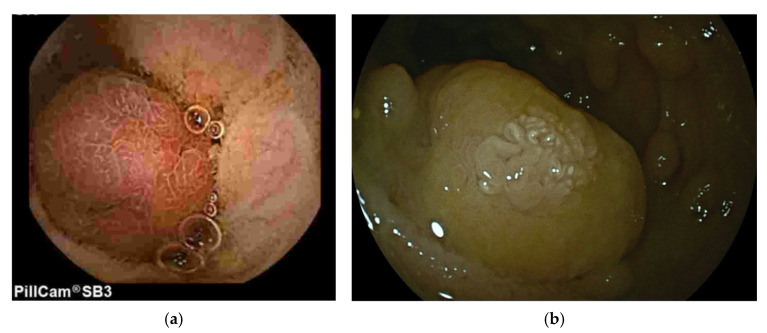
Large ileal Peutz-Jeghers polyp identified during small bowel capsule endoscopy (PillCam *SB3*) (**a**), and (**b**) referred for resection by retrograde double-balloon enteroscopy.

Peutz-Jeghers syndrome (PJS) is an inherited autosomal dominant polyposis syndrome with characteristic melanin mucocutaneous pigmentations along with hamartomatous polyps found in the gastrointestinal tract, including the jejunum and ileum [7,30,42,43,44]. Peutz-Jeghers polyps are characterized by a distinctive arborizationarborisation of smooth muscle within the lamina propria, a characteristic not found in adenomatous polyps [45]. Small bowel capsule endoscopy is important for small bowel surveillance and detection of clinically significant (>15 mm) polyps. Contrary to previous belief, large PJS polyps are not thought to be associated with malignant transformation and risk of small bowel malignancy. However, they are associated with a risk of intussusception and small bowel obstruction. A Dutch study of 110 patients with PJS showed a high cumulative risk of intussusception early in life (50% at the age of 20 years). In all, 76 patients (69%) had experienced at least one intussusception. The intussusceptions were generally caused by hamartomas ≥ 15 mm in diameter, with polyp size being the most important risk factor for small-bowel intussusception. The exact size of the intussusception-causing polyp could be determined in 37 events. These intussusceptions had been caused by hamartomas with a median size of 35 mm (range 15–60 mm). Of these 37 hamartomas, 3 were < 20 mm (15, 18, and 18 mm, respectively) [46]. Therefore, large or symptomatic polyps should be selected for prophylactic endoscopic resection to prevent intussusception. Representative images of a large PJS polyp as seen at VCE with its corresponding image at double-balloon enteroscopy performed for endoscopic resection of the polyp are shown in Figure 1.

The polyp burden in a paediatric population was assessed in a 10-year retrospective longitudinal analysis using SBCE (PillCam *SB2* or *SB3*) by Steward et al. [47]. The cohort included 15 patients diagnosed with PJS and a total of 61 SBCE reports. The authors showed that fewer than 30 polyps with a size smaller than 20 mm were found in 57 SBCE. In 4 SBCE, polyps were greater than 21 mm in size. Polyp distribution was also studied with 77% of patients identified with proximal polyps, and 66% in the distal small bowel.

SBCE polyp surveillance was also evaluated in an observational study with 14 patients with PJS and 6 symptomatic relatives with first-degree PJS using *SB2* or *SB3* [11]. In the PJS group, more polyps were found in the jejunum compared to duodenum and ileum. Polyps larger than 11 mm were detected in seven (50%) patients; five of these patients underwent enteroscopy, which revealed that capsule endoscopy had correctly identified all the patients with polyps over 11 mm, but had missed 20% of the total number of large polyps. In the second group, no polyps were detected by *SB2*/*3*, avoiding the need for further investigation.

These results are particularly important in the paediatric population, where the risk of intussusception is high, and where SBCE may play a preventive role [7,35]. By the age of 10 approximately 30% of patients have developed symptoms, and 50% by the age of 20. Data from the St Mark’s polyposis registry demonstrated that 68% of adults with PJS had undergone a previous laparotomy by the age of 18 [48].

According to the ESGE guidelines [49] and the latest European Hereditary Tumour Group (EHTG) [50] guidelines, either SBCE or magnetic resonance enterography (MRE) is recommended at the age of 8 [35]. The choice of imaging modality depends on local expertise. In addition, the developmental age of children should be taken into account, since patients need to remain still during the MRE examination [35] and SBCE may be preferable in younger patients [51,52]. However, it is worth considering that very young patients may not be able to swallow the capsule, and in those cases endoscopic placement under general anaesthesia may be required. This may be performed at the time of their regular upper GI surveillance. However, for those patients with no polyps at baseline who would normally require their next surveillance gastroscopy at the age of 18, small bowel surveillance with capsule endoscopy would require additional GA procedures for capsule placement. In these cases, MRI small bowel may avoid the need for additional GA procedures. If SBCE is selected for PJS small bowel polyp surveillance, the procedure is repeated every 3 years [42].

Polyp detection rates using SBCE in patients with PJS range between 75% and 90% [12,20]. Numerous studies have compared SBCE to other imaging modalities [53], though older techniques such as small-bowel follow-through are no longer routinely used in clinical practice [54]. In a blinded comparison study by Postgate et al. [9], which involved children with PJS undergoing both *SB1* and barium enterography, *SB1* identified significantly more polyps than barium enterography (*p* = 0.02). However, no significant difference was observed in the detection of larger polyps (>10 mm) (*p* = 0.50). Questionnaire data revealed that 90% of children preferred *SB1* (*p* = 0.02), considering it more comfortable than barium enterography (*p* = 0.03).

A similar study conducted in adults with PJS demonstrated that *SB1* detected a greater number of polyps larger than 10 mm compared with barium studies (*p* = 0.008), with most participants expressing a preference towards capsule endoscopy for future surveillance [55]. Additionally, in an observational report by Burke et al. [8], *SB1* was compared with small bowel radiography in four adult patients with post-colectomy PJS. In two of these cases, *SB1* revealed diffuse polyposis, whereas radiographic findings were normal. In another patient, *SB1* detected over 20 diffuse small bowel polyps exceeding 10 mm, while radiography identified only 1 polyp under 1 cm in the proximal jejunum. Notably, *SB1* led to a change in management in 50% of patients with PJS by enabling the identification of small bowel polyps, which prompted subsequent surgical polypectomy [2,20].

Additional studies incorporating direct visualisation methods have contributed to the comparative evaluation of SBCE. Ohmiya et al. [10] conducted a retrospective analysis of 18 PJS patients, comparing *SB1* with fluoroscopic enteroclysis and double-balloon enteroscopy (DBE). *SB1* achieved similar polyp detection rates to DBE, while fluoroscopic enteroclysis identified fewer lesions. In a prospective study by Schulmann et al. [7], 11 patients with non-obstructive PJS were assessed using MRE, push enteroscopy, and *SB1*. The cohort was divided into symptomatic and asymptomatic groups. Among symptomatic individuals, both MRE and *SB1* effectively detected distal ileal polyps, while push enteroscopy failed to detect a large lesion. In the asymptomatic group, *SB1* revealed jejunal or ileal polyps in four patients that went undetected by push enteroscopy. MRE also missed distal jejunal polyps < 2 cm in two cases. Importantly, intraoperative enteroscopy following surgical resection did not identify additional polyps beyond those already visualised by *SB1*, further validating its diagnostic sensitivity. Collectively, these studies underscore the high diagnostic yield and patient acceptability of capsule endoscopy in PJS surveillance. While newer imaging and endoscopic techniques like MRE and device-assisted enteroscopy offer complementary information, SBCE remains a minimally invasive, well-tolerated option with strong performance, particularly for detecting clinically relevant small bowel polyps. Nonetheless, its limitations in precise localisation and the potential for missed lesions in certain anatomical regions suggest that multimodal evaluation may be appropriate in selected cases.

In an Australian prospective study, 20 PJS patients underwent both *SB1* and MRE for polyp surveillance [56]. *SB1* identified significantly more polyps ≥ 10 mm than MRE (47 vs. 14, *p* = 0.02) and detected at least one significant polyp in 55% of patients compared to 35% with MRE (*p* = 0.25). However, the concordance between the two modalities was limited, with only 40% agreement (8/20) regarding the presence or absence of significant lesions. Subsequent balloon-assisted enteroscopy performed in 12 patients confirmed a total of 26 significant polyps in eight individuals. When compared with balloon enteroscopy, MRE showed a higher agreement (75%) than *SB1* (50%) and demonstrated a superior positive predictive value (100% vs. 60%, respectively). These findings highlight that although *SB1* may detect a higher number of polyps, especially smaller lesions, it also carries a risk of overestimating or mislocalising polyps, thereby underscoring the need for cautious interpretation and, where possible, correlation with other modalities.

Gupta et al. [12] evaluated *SB2* in a prospective study comparing its performance against MRE in 19 PJS patients with PJS [34]. *SB2* demonstrated a significantly improved detection rate for polyps smaller than 10 mm (*p* = 0.03). For polyps larger than 10 mm, the difference in detection between *SB2* and MRE was not statistically significant (*p* = 0.35). Notably, *SB2* missed three polyps exceeding 15 mm in size (*p* = 0.18), which raises concern about its reliability in identifying the most clinically relevant lesions. These findings suggest incremental improvements in polyp detection with *SB2*, particularly for smaller lesions, due to the improved technical characteristics of *SB2* compared with *SB1*, but limitations remain regarding missed lesions, presumably because these are often due to rapid capsule transit or blind spots. A summary of the comparative studies between VCE and alternative imaging techniques is provided in Table 3.

Taken together the studies of capsule endoscopy in PJS demonstrate the value of SBCE in small bowel polyp surveillance for the detection of clinically significant polyps (≥10–15 mm) that are generally associated with a higher risk of intussusception and should be selected for polyp resection by deep enteroscopy. SBCE appears to be well tolerated and has better diagnostic accuracy for clinically significant polyps compared with historical imaging modalities (such as barium contrast studies) and similar performance to MRE. Many of the published reports have used older generation SBCE systems (*SB1* or *SB2*) and more studies using the latest PillCam *SB3* and systems from other manufacturers (such as MiroCam, CapsoCam, etc.) would be useful to more accurately determine the performance metrics of capsule endoscopy. Missed lesions remain an inherent limitation of SBCE due to rapid transit or blind spots, particularly in the duodenum and proximal small bowel, and imaging techniques are often used as a complementary tool for small bowel surveillance.

### 3.2. Familial Adenomatous Polyposis (FAP)

Familial adenomatous polyposis (FAP) is another inherited autosomal dominant syndrome affecting both the small and large intestine. Without intervention the estimated risk of colorectal cancer is 100% [33]. Duodenal adenomas are found in 80% of FAP cases [33,57]. At St Mark’s Hospital, unpublished data suggest that the lifetime risk of non-duodenal small bowel cancer is diminutive. In over 100 years in our registry, only 5 cases of non-duodenal small bowel cancer have been identified. Of these cases, 2 involved cancers had arisen on the stoma spout. This of course isa distinct scenario given the continuous exposure to effluent and the ease with which adenomas can be detected by the patient, making VCE of little value in such cases. The remaining 3 cases were jejunal cancers.

The Spigelman classification is commonly used to record the severity of polyposis in the duodenum, based on points given by the number of polyps, polyp size, polyp histology, and presence of dysplasia (Table 4) [7]. In the duodenum, stage 4 Spigelman occurs in 10% of FAP patients post-polypectomy [7], and periampullary carcinoma, detected in 4% of cases, is the main cause of cancer in this subcategory [33].

In a prospective study of 14 FAP patients who underwent SBCE with *SB1*, the ampulla was not visualised in any of these cases [58]. Regardless of the presence of duodenal polyps, jejunal or ileal polyps were identified by *SB1* in 30–60% of FAP patients [7,33]. In a study by Günther et al. [59], 13 of the 15 (87%) FAP patients had small-bowel polyps detected by *SB1* with estimated size ranging from <5 mm to >10 mm. In four of these patients, medium-sized (5–10 mm) or large (>10 mm) polyps were seen, all located in the proximal jejunum.

The British Society of Gastroenterology (BSG) guidelines recommends surveillance with conventional forward viewing gastroscopy combined with the use of a side-viewing endoscope in asymptomatic patients starting at the age of 25 [35,38,60]. The polyp detection rate of VCE tends to be lower for polyps found in the proximal small bowel, including the duodenum and ampulla of Vater, due to the rapid transit of the capsule through the proximal small bowel.

In a previous study of patients with FAP, *SB1* reported only 36.4% of the duodenal polyps that were detected at endoscopy [33]. When duodenal adenomas are detected during gastroduodenoscopy, further polyps may be discovered in the distal ileum, although their clinical significance is uncertain [7,61].

In a study by Iaquinto et al. [33], although *SB1* failed to detect most duodenal polyps identified by gastroscopy, it successfully revealed a considerable number of jejunal and ileal polyps (<5 mm) in seven patients. The presence of duodenal adenomas was the only predictor of distal small bowel involvement, suggesting *SB1* may serve as a complementary modality for assessing post-duodenal disease.

Matsumoto et al. [62] studied the utility of *SB2* in FAP patients post-colectomy. Among 39 patients, small bowel polyps 1–5 mm in diameter were found in 20 cases. The majority were found in the jejunum, with ileal polyps identified in four cases. Given the high completion rate of *SB2* (92.9%) and the lack of adverse events, including capsule retention, it was suggested that surveillance SBCE can be safely conducted in post-colectomy FAP patients.

In another comparative prospective study reported by Akin et al. [63], six FAP patients underwent both *SB1* and MRE. Four patients had polyps ≤ 10 mm, seen only by *SB1*, and cross-sectional imaging with MRE identified extraintestinal desmoid tumors. The authors concluded that in patients with FAP, *SB1* can detect small-sized polyps in the small intestine which may be missed by MRE.

Tescher et al. [64] compared *SB1* with MRE, small bowel barium follow-through (SBFT) and side-viewing gastroscopy in 20 patients with FAP. The study showed that *SB1* was the only imaging modality that identified polyps in all bowel segments, demonstrating a significantly higher total number of polyp findings in the jejunum, ileum and caecum than MRE and SBFT. For all regions the majority of polyps diagnosed by *SB1* were <6 mm. Overall, gastroscopy identified most gastric and duodenal findings, and *SB1* most findings beyond the duodenum. In 13 patients, gastroscopy detected more duodenal polyps than the other three procedures and either the same or fewer than *SB1* in 6 patients (*p* < 0.001).

Plum et al. [65] evaluated prospectively multiple imaging modalities in 25 FAP patients known to have duodenal adenomas. Push enteroscopy, *SB1*, ileoscopy and enteroclysis were used for small bowel assessment. In 22 of the 23 patients who underwent *SB1*, small bowel adenomas were observed. In total, 13 of these 22 patients also had adenomas in regions not accessible to push enteroscopy or ileoscopy. The adenoma detection rate was low for enteroclysis (17%) with 19 false-negative results (adenomas), eight of which were ≥10 mm. The authors suggested that *SB1* is a safe and convenient method for evaluating the small bowel in these patients, while enteroclysis is inferior to the endoscopic procedure’s evaluation of the small bowel in FAP.

However, findings by Wong et al. [66] suggested that the polyp burden may be underestimated by *SB1*. Their team compared the polyp detection rates of *SB1*, push enteroscopy and lower endoscopy in 32 patients with FAP. A defined small bowel segment proximal to a previously placed tattoo within the proximal small bowel was assessed. In addition, the entire small bowel was examined in patients with a high number of proximal small bowel polyps. More polyps were identified by push enteroscopy in the defined small bowel segment proximal to the tattoo (*p* = 0.002). Compared with *SB1*, the combination of push enteroscopy and lower endoscopy also detected significantly more polyps throughout the entire examined small bowel (*p* < 0.001). The authors concluded that in patients with FAP, *SB1* underestimates the number of small bowel polyps. Table 5 summarises the comparative studies in FAP.

Regarding colon capsule endoscopy in FAP, Cavallo et al. [69] performed a retrospective evaluation of *CCE-1* and *CCE-2* used as the first screening examination for the surveillance of the upper and lower gastrointestinal tract in 14 adolescents. The CCE study was completed in 13 patients (93.3%). CCE identified the duodenal papilla in 4 patients and colonic and rectal polyps in all 13 patients. The authors reported that the procedure was feasible and well tolerated as a first screening examination in adolescents with FAP. They concluded that although CCE should not be used as an alternative to colonoscopy, it could improve compliance with colonoscopy and increase early adherence to a surveillance program.

Comparative studies have also been conducted evaluating the diagnostic yield of small bowel capsule compared with radiological examinations in a mixed hereditary polyposis population. Caspari et al. [67] performed an observational study comparing the diagnostic yields of MRE and *SB1* for the detection of small bowel polyps. Four patients with PJS and sixteen with FAP underwent MRE and *SB1*. In total, 448 polyps, with sizes ranging between 1 mm and 30 mm were detected by *SB1*, while only 24 polyps, all over 5 mm, were identified by MRE. In this study, *SB1* and MRE detection rates for polyps larger than 15 mm were similar, whereas smaller polyps were seen more often with *SB1* and polyps smaller than 5 mm were exclusively seen with *SB1*. Polyp localisation and size estimation were, however, more precise with MRE. *SB1* has also been compared with small bowel follow-through [68] in 24 patients with polyposis syndromes, 20 diagnosed with FAP, and 4 with PJS. Small bowel polyps were detected in 7 of 24 patients by *SB1*, while a barium contrast study identified small intestinal polyps in only 3 of the 7 patients. In the four remaining patients, all with FAP, polyps detected by *SB1* but not reported in radiographic series were located in the ileum (two out of four), jejunum (one), and duodenum (one). The study concluded that *SB1* is an accurate test for the detection of small bowel polyps in hereditary polyposis syndromes.

In summary, most guidelines do not recommend routine use of VCE in FAP patients, as small bowel polyps in the jejunum and ileum do not appear to be associated with progression to cancer [70]. The reports of capsule endoscopy in FAP highlight the known limitations of SBCE in the assessment of the duodenum and ampullary region, where most small bowel cancers occur. The rapid transit of the capsule through the duodenum and angulations of the duodenal segment resulting in blind spots are usually responsible for these limitations, and gastroscopy with forward and side viewing examination should be used for surveillance of the duodenum. In cases of carcinoma development, positive resection margins after endoscopic polypectomy, or when surveillance is not feasible, Whipple’s procedure may be considered [71,72,73,74,75].

### 3.3. Juvenile Polyposis Syndrome (JPS)

Juvenile polyposis syndrome (JPS) belongs to the autosomal dominant hamartomatous polyposis syndromes involving variants in the SMAD4 and BMPR1A genes. JPS is associated with hamartomatous polyps in the colon (98%), stomach (14%), jejunum and ileum (7%) and duodenum (7%). The diagnosis is certain when one or more of the following criteria are met: more than five juvenile polyps in the colon or rectum, multiple juvenile polyps throughout the gastrointestinal tract, or any number of juvenile polyps with a family history of JPS. JPS-associated polyps are less likely to require removal due to the low risk of cancer development and bowel obstruction [35,76]. A case series study by Postgate et al. [77] showed that *SB1* identified two of 10 patients diagnosed with JPS who had small bowel polyps beyond the reach of standard gastroscopy, while a third of patients had duodenal polyps revealed only at *SB1*.

Routine small bowel capsule polyp surveillance is not recommended in JPS patients. However, in anaemic patients and in cases of SMAD4 variants, SBCE is proposed for evaluation of Hereditary Haemorrhagic Telangiectasia (HHT) overlap syndrome [36,78].

There is a small group of patients with microdeletion in chromosome 10 which affect PTEN and BMPRIa [79]. These have a particularly severe phenotype which includes protein losing enteropathy, hypoalbuminaemia and anaemia. There are no data about the utility of VCE in this patient subgroup.

### 3.4. Phosphatase and Tensin Homolog Hamartoma Tumour Syndrome (PHTS)

Phosphatase and tensin homolog hamartoma tumour syndrome (PHTS) is a rare autosomal dominant polyposis syndrome caused by *PTEN* gene variants. The diagnosis consists of major criteria (breast cancer, endometrial cancer, thyroid cancer, gastrointestinal hamartomas, Lhermitte-Duclos disease, macrocephaly, macular pigmentation of the glans penis, mucocutaneous lesions including trichilemmomas and acral keratoses) and minor criteria (autism, colon cancer, oesophageal glycogenic acanthosis, lipomas, renal carcinoma, testicular lipomatosis, thyroid cancer and vascular abnormalities) [76].

The phenotype also includes the presence of polyps in the small bowel, especially in the duodenum and jejunum [80]. Patients with PHTS may develop small bowel polyps but there is no clear evidence that there is an increased risk of SB cancer in this patient group. There are no data to suggest that routine SB surveillance provides any benefit to patients and routine VCE surveillance is not included in the latest PTEN guidelines [81,82].

Even in symptomatic patients with PHTS, the role of VCE is not well established. There are reports about symptomatic children with PHTS who underwent SBCE as part of their diagnostic evaluation [18]. In a case series of 10 patients with PHTS, 90% of *PTEN* mutation positive patients had duodenal polyps identified during participation in an annual upper and lower endoscopy surveillance programme. These were mostly hyperplastic and hamartomatous, but three patients also had adenomatous duodenal polyps [37]. Of interest, Hatogai et al. [83] showed that contrast image *SB1* using a flexible spectral imaging colour enhancement system did not affect the diagnostic accuracy or number of detected polyps in six patients with polyposis syndromes (FAP, Cowden syndrome, and Cronkhite–Canada syndrome), but hamartomatous polyp micro-structures were more clearly visualised on contrast than pre-contrast images.

### 3.5. Other Polyposis Syndromes (MAP, PPAP, NAP)

*MUTYH*-associated polyposis (MAP) belongs to the autosomal recessive syndromes and mainly affects adult patients. Its pathogenesis involves bi-allelic *MUTYH*-variants and it is associated with cancer development in the colon, and also the duodenum and small bowel [38]. To the best of our knowledge no studies have been published on the role of VCE in MAP. Therefore, VCE is not routinely suggested.

Inherited deficiencies in DNA repair mechanisms underlie several genetic cancer predisposition syndromes, which often manifest with adenomatous polyposis and extraintestinal tumours. These include polymerase proofreading-associated polyposis (PPAP)—an autosomal dominant syndrome caused by pathogenic mutations in the proofreading domains of the *POLE* and *POLD1* genes [40]—and autosomal recessive disorders linked to defects in base excision repair (BER) genes like *NTHL1*. The latter leads to *NTHL1*-associated polyposis (NAP), an autosomal recessive syndrome linked to an increased risk of colorectal and extraintestinal malignancies [41]. As per the latest BSG guidelines [38], evidence remains limited regarding surveillance strategies for these rare syndromes. The use of VCE has not been studied in these syndromes.

## 4. Discussion

In current practice, VCE serves primarily as a non-invasive first-line modality for mapping small-bowel polyp burden and identifying lesions requiring therapeutic intervention. It is particularly valuable in asymptomatic PJS patients, in whom capsule findings guide timing and route of device-assisted enteroscopy [35]. Conversely, interventional or cross-sectional techniques are preferred for targeted therapy, biopsy, or when luminal obstruction or ampullary pathology is suspected, as in advanced FAP [66]. Combining modalities—VCE for detection and enteroscopy for resection—remains the most effective strategy for comprehensive small-bowel assessment.

The diagnostic role of VCE is best established in PJS, where its sensitivity for jejuno-ileal polyps ≥ 10–15 mm and excellent tolerability make it the preferred surveillance tool [46] alongside MRE [35]. In contrast, its role in FAP remains adjunctive; VCE may detect jejunal and ileal adenomas [33] but is limited in assessing the duodenum and ampulla, where conventional side-viewing endoscopy remains essential [33]. For JPS, PHTS, and rarer entities such as MAP, PPAP, and NAP, data are sparse and largely derived from small observational series or case reports. In these settings, VCE may assist in symptomatic evaluation or research protocols but is not routinely recommended for surveillance [38,78,81,82]. This spectrum of evidence highlights that while VCE’s diagnostic potential spans multiple syndromes, its established clinical utility currently lies predominantly in PJS.

Despite its advantages, SBCE has a false negative rate, including for larger polyps [2]. SBCE may fail to detect adenomas in the proximal small bowel due to the low frame rate of small bowel capsule (four frames per second, FPS), which is too low to compensate for its rapid transit in that area. The miss rate of small bowel tumours has been estimated to be as high as 18.9%. When SBCE was compared with duodenoscopy in 145 patients, 2 out of 15 tumours were not diagnosed [30,84]. However, colon capsule endoscopy with a high frame rate of up to 35 FPS appears to achieve excellent rates of polyp detection in complete and adequately prepared examinations. A prospective study by Rex et al. of 884 asymptomatic patients who had CCE-2 followed by ileo-colonoscopy revealed the sensitivity and specificity of CCE-2 was 81% and 93% respectively for polyps ≥ 6 mm, and 80% and 97%, respectively, for polyps ≥ 10 mm. For adenomas ≥ 6 mm, the sensitivity and specificity of CCE-2 were 88% and 82% respectively, and for adenomas ≥ 10 mm, 92% and 95% respectively [85]. Similar results were shown in a subsequent study by Pecere et al. [86] which demonstrated a good sensitivity (90%) of CCE-2 in the detection of polyps with advanced neoplasia ≥ 6 mm in a screening population with positive fecal immunochemical test (FIT). A recent observational study by Turvill et al. [87] compared CCE-2 with colonoscopy and CT-colonography among symptomatic adults with FIT ≤ 100 μg Hb/g. Among 10,369 patients, 4878 underwent CCE, 5025 colonoscopy and 466 CT colonography. When the examination was both complete and of adequate quality, CCE successfully identified a corresponding mass lesion in all patients diagnosed with colorectal cancer. Polyp detection rates for lesions larger than 10 mm and for those measuring 6–9 mm were higher with CCE compared with either colonoscopy or CT colonography. In patients with a paired, complete, and adequately prepared CCE and colonoscopy, the per-patient sensitivity for detecting polyps ≥ 10 mm and 6–9 mm was 97%. Nonetheless, a significant limitation was that the CCE completion rate (74%) and adequacy of bowel preparation (74%) were lower than those achieved with colonoscopy and CTC (both 88%). However, CCE usefully performed a filter function in 86% of patients and effectively demonstrated its colonoscopy sparing capacity.

Another limitation of the small bowel capsule is that the estimation of polyp size may be inaccurate, and unlike the colon capsule, the SBCE software (i.e., PillCam™ version 9.0, Medtronic, Dublin, Ireland [88]) does not provide a polyp size estimation tool. Of interest, a study by Rácz et al. [89] evaluated the measurement of polyp sizes in PJS patients using reference granules during capsule endoscopy with *SB1*. Patients ingested one sachet of Salofalk^®^ Granu-Stix^®^ consisting of 600 granules, each of 1 mm diameter in size, 5 min prior to the procedure. The real size of small bowel polyps could be accurately evaluated by comparing the polyps with adjacent granules using a simple mathematical equation. The granules dissolved in the distal ileum and did not affect the image quality. The correct estimation of polyp size was verified in 3 patients post-polypectomy by enteroscopy.

In addition, in patients with surgically altered anatomy, such as those who have undergone Whipple procedures or Roux-en-Y reconstructions, capsule endoscopy may fail to evaluate the afferent limb. In such cases, cross-sectional imaging may be required to assess the excluded bowel segments.

Finally, one of the limitations of small bowel capsule endoscopy is the accuracy of polyp localisation. Although transit time and progress-based indices are commonly used for localisation purposes, these provide only an estimation of the polyp site [35]. The latter could also be distorted due to capsule hold up in certain parts of the small bowel, most commonly in the duodenum and terminal ileum. MRE may be more accurate for determining polyp location in the small bowel, and is often used subsequent to SBCE to aid the localisation of large polyps, especially when intervention is needed [67].

## 5. AI and Future Perspectives

Artificial intelligence (AI) has shown promise in the video capsule field. VCE can generate more than 50,000 images, therefore reading and reporting is time-consuming and prone to error task. Neural networks and deep learning could be leveraged to expedite and enhance the accuracy of VCE readings [90]. A recent large-scale study involving 10^8^ images from 77 medical centres demonstrated that a neural network algorithm can outperform gastroenterologists in detecting small-bowel lesions, including polyps, with a sensitivity of 99.9% compared with 76.89% (*p* < 0.001), while drastically reducing the reading time from an average of 96.6 ± 22.53 min to 5.9 ± 2.2 min [91].

A promising study has been published by Deding et al. [92] regarding the use of AI in CCE-2 after incomplete colonoscopy. The authors used an AI-based localisation algorithm to estimate the location of capsules and any pathological abnormality to be removed or biopsied in the colon (rectum, left colon, transverse colon, right colon). The model output was compared with the physician assessment. Overall, the agreement was found to have a mean value of 77% and a median of 85%. The authors suggested that the improvement of AI-based localisation of capsules using a forward-tracking algorithm may increase the rate of complete colonic mucosal visualisation in individuals with incomplete colonoscopy, by identifying the point of maximal insertion at incomplete colonoscopy, thereby completing the colonoscopic assessment even in cases when the CCE is incomplete too (if it does not reach the haemorrhoidal cushions or is not excreted).

So far, there are no studies in the literature regarding AI in VCE for patients with polyposis syndromes. Future research should focus on the use of AI in detection, localisation and accurate polyp number estimation in cases of polyp multiplicity. Nanotechnology incorporating nanorobots, already being tested in vivo in animal models, could also have dual effect in theranostics in the future [93].

Finally, the use of panenteric capsule endoscopy as a minimally invasive evaluation of the entire gastrointestinal tract may also be a promising area for future research in polyposis, particularly when evaluation of the stomach, small bowel, and colon is required, such as in PJS patients. In these cases, it would provide the potential option to assess the whole gastrointestinal tract in a single procedure without the need of invasive diagnostic procedures. The panenteric capsule is already widely used in Crohn’s disease assessment, providing a panintestinal assessment of the disease extent and severity, and is part of the recently published BSG guidelines [94]. In a multicentre study [95], panenteric VCE was carried out in 249 procedures involving both adults and children with Crohn’s disease, resulting in therapeutic adjustments in 196 cases. VCE is also utilised in the evaluation of iron deficiency anaemia [96].

Beyond technical advances, health-economic aspects warrant further investigation. Comparative cost-effectiveness studies assessing VCE against conventional radiologic surveillance across healthcare systems could define its cost-effectiveness in routine practice. Current data show that VCE cost is considerable higher [97,98] than magnetic resonance enterography (MRE) [99] or computed tomography enterography (CTE) [100]. Incorporating patient adherence, diagnostic yield, and resource utilisation metrics into future analyses would strengthen the rationale for broader clinical adoption.

## 6. Limitations

This is a narrative, non-systematic review without a formal risk-of-bias assessment, or conduct quantitative synthesis. Although the search was structured, it was limited to PubMed and Scopus and to English-language publications, introducing potential selection and language bias. The literature mostly includes observational, prospective studies with only a few retrospective studies [10,47,69] or case series [77], while randomised trials are lacking. Evidence across syndromes is also heterogeneous, with older studies using earlier-generation capsules (*SB1*/*SB2*), which may not reflect current device performance. For rarer syndromes (e.g., MAP, PPAP, NAP), data are sparse or absent, limiting generalisability. Size estimation and lesion localisation issues reported in included studies further constrain comparative interpretation. These factors should be considered when interpreting our synthesis.

## 7. Conclusions

VCE represents a complementary tool in the diagnosis, surveillance and management of polyposis syndromes. Its application is most established in PJS where it offers an effective, minimally invasive tool for the detection and surveillance of clinically significant small bowel polyps that may require prophylactic therapeutic intervention. SBCE is often then combined with MRE when significant sized polyps are identified, to aid in the accurate sizing and localisation of lesions. A few studies have also investigated the role of VCE in other polyposis syndromes, such as FAP by detecting small bowel polyps, although it has limitations in visualising the duodenum where most cancers occur. In addition, small bowel polyp progression to cancer is a rare event and therefore routine capsule surveillance is not recommended. Similarly, in JPS, while VCE is not routinely recommended, it can be useful in anaemic patients or those with *SMAD4* variants to investigate the possibility of HHT overlap.

With the advent of new capsule endoscopes that have been developed to specifically assess the colon or offer pan-enteric evaluation, the role of capsule endoscopy in polyposis syndromes requires further research. Despite its advantages, VCE has several limitations, including inability to perform biopsies, and the potential for false negative and false positive findings. Future advances, particularly in AI integration and the development of therapeutic capsules, appear promising, and these innovations could transform VCE into a more powerful diagnostic and therapeutic tool for the management of polyposis patients.

## Figures and Tables

**Table 1:** Small bowel capsule endoscopy types from different manufacturers and their year of release to the market.

Capsule Name	Manufacturer	Year Released
PillCam SB [7,8,9,10]	Given Imaging Ltd. (Yokneam, Israel)	2001
EndoCapsule [10]	Olympus (Tokyo, Japan)	2005
Pillcam *SB2* [11,12]	Medtronic (Minneapolis, MN, USA)	2007
OMOM Capsule [13]	Jinshan Science & Technology (Chongqing, China)	2008
Capsocam SV-1 [14]	Capsovision (Saratoga, NY, USA)	2011
PillCam *SB3* [3]	Medtronic (Minneapolis, MN, USA)	2013
MiroCam MC1000 [15]	Intromedic (Seoul, Republic of Korea)	2014
Endocapsule 10 [2]	Olympus (Tokyo, Japan)	2014
Capsocam SV-2 [16]	Capsovision (Saratoga, NY, USA)	2014
Mirocam MC1200 [17]	Intromedic (Seoul, Republic of Korea)	2015
OMOM Capsule 2 [13]	Jinshan Science & Technology (Chongqing, China)	2018
MiroCam MC 1600 [18]	Intromedic (Seoul, Republic of Korea)	2018
Mirocam MC2000 [19]	Intromedic (Seoul, Republic of Korea)	2018
CapsoCam Plus [5]	Capsovision (Saratoga, NY, USA)	2019
OMOM HD [6]	Jinshan Science and Technology (Yubei, China)	2020
MiroCam 4000 [4]	Intromedic (Seoul, Republic of Korea)	2020
NaviCam SB with ProScan™ [20]	AnX Robotica (Plano, TX, USA)	2024

**Table 2:** Polyposis syndromes, characteristics and VCE guidelines.

Polyposis Syndrome	Inheritance Pattern	Polyp Type	Small Bowel Involvement	Use of VCE for Surveillance	Additional Comments
PJS	Autosomal dominant [7]	Hamartomatous [7]	Invariably [30]	Start at the age of 8 and repeat every 3 years [31].	VCE versus MRE: a. VCE better in the detection of jejuno-ileal polyps less than 10 mm [30] b. VCE may miss larger polyps [32]
FAP	Autosomal dominant [33]	Adenomatous [33]	80%, especially in duodenum [33]	Not routinely [2].	N/A
JPS	Autosomal dominant [34]	Juvenile [34]	7% in jejunum, ileum or duodenum [34]	Not routinely (Only in symptomatic patients and in those with SMAD4 variants) [35].	VCE suggested to rule out HHT [36].
PTEN-HS	Autosomal dominant [34]	Hyperplastic/Hamartomatous/Juvenile/Adenomatous [34]	90% duodenum [37]	Not routinely [35].	N/A
MAP	Autosomal recessive [38]	Adenomatous [38]	Duodenal involvement 34% [39]	Not routinely [38].	N/A
PPAP	Autosomal dominant [40]	Adenomatous [40]	N/A (association with colorectal cancer) [40]	N/A	N/A
NAP	Autosomal recessive [41]	Adenomatous [41]	N/A (association with colorectal cancer) [41]	N/A	N/A

**Table 3:** Comparative studies in PJS.

Author	Study Design	Year	Population	No of Patients	Capsule Type	Comparator	Outcome	Reference
Postgate et al.	Prospective	2009	Paediatric	11	*SB1*	Barium enterography	More polyps were found by *SB1* compared with barium enterography (*p* = 0.02), but there was no difference for polyps larger than 10 mm.	[9]
Brown et al.	Prospective	2006	Adults	19	*SB1*	Barium enterography	*SB1* detected more polyps larger than 10 mm when compared with barium studies (*p* = 0.008).	[55]
Burke et al.	Prospective	2005	Adults post-colectomy (mixed population with PJS/FAP)	3 with PJS	*SB1*	SB radiography	In two cases, *SB1* revealed diffuse polyposis while radiography was normal. In another patient, *SB1* detected more than 20 diffuse small bowel polyps larger than 10 mm, but only 1 polyp (less than 1 cm) was found during radiography [8].	[8]
Ohmiya et al.	Retrospective	2010	Adults	18	*SB1*	Fluoroscopic enteroclysis/ Double-balloon enteroscopy	*SB1* polyp detection rates were similar to enteroscopy, while less polyps were found by enteroclysis	[10]
Schulmann et al.	Prospective	2005	Adults	11	*SB1*	MRE/Push enteroscopy	Among symptomatic individuals, both MRE and *SB1* detected distal ileal polyps, while push-enteroscopy failed to detect a large lesion. In the asymptomatic group, *SB1* revealed jejunal or ileal polyps that went undetected by push-enteroscopy in four patients.	[7]
Urquhart et al.	Prospective	2014	Adults	20	*SB1*	MRE	The total number of polyps ≥ 10 mm detected by *SB1* was greater compared with MRE.	[56]
Gupta et al.	Prospective	2010	Adults	19	*SB2*	MRE	*SB2* detected more polyps smaller than 10 mm compared with MRE (*p* = 0.03).	[12]

**Table 4:** Spigelman classification [7]. Stage 0 = 0 points, stage 1 = 1–4 points, stage 2 = 5–6 points, stage 3 = 7–8 points, stage 4 = 9–12 points.

Criterion	Points		
	1	2	3
Polyp number	1–4	5–20	>20
Polyp size (mm)	1–4	5–10	>10
Histology	Tubular	Tubulovillous	Villous
Dysplasia	Mild	Moderate	Severe

**Table 5:** Comparative studies in FAP.

Author	Study Design	Year	Population	No of FAP Patients	Capsule Type	Comparator	Outcome	Reference
Caspari et al.	Prospective	2004	Mixed patients (FAP/PJS)	16 FAP	*SB1*	MRE	*SB1* and MRE detection rates for polyps larger than 15 mm were similar, whereas smaller polyps were seen more often with *SB1* and polyps smaller than 5 mm were exclusively seen with *SB1*.	[67]
Akin et al.	Prospective	2012	Only FAP	6	*SB1*	MRE	Four patients had polyps ≤ 10 mm, seen only by *SB1*.	[63]
Mata et al.	Prospective	2005	Mixed patients (FAP/PJS)	20 FAP	*SB1*	SB follow-through	In 4 FAP patients, polyps detected by *SB1* but not reported in radiographic series.	[68]
Tescher et al.	Prospective	2010	Only FAP	20	*SB1*	MRE/SBFT/Side-viewing gastroscopy	*SB1* was the only imaging modality that identified polyps in all bowel segments, demonstrating a significantly higher total number of polyp findings in the jejunum, ileum and caecum than MRE and SBFT.	[64]
Plum et al.	Prospective	2009	Only FAP	25	*SB1*	Push enteroscopy/ Ileoscopy/ Enteroclysis	Thirteen of the patients had adenomas in regions not accessible to push enteroscopy or ileoscopy. *SB1* was a safe and convenient method for evaluating the small bowel in these patients, while enteroclysis wasinferior to the endoscopic procedures for evaluation of the small bowel in FAP.	[65]
Wong et al.	Prospective	2006	Only FAP	32	*SB1*	Push enteroscopy /Ileocolonoscopy	Compared to *SB1*, the combination of push enteroscopy and lower endoscopy also detected significantly more polyps throughout the entire examined small bowel (*p* < 0.001).	[66]

## Data Availability

No new data were created.

## Abbreviations

The following abbreviations are used in this manuscript:

AI	Artificial Intelligence
BMMRD	Biallelic Mismatch Repair Deficiency
BSG	British Society of Gastroenterology
CCE	Colon Capsule Endoscopy
CTE	Computed Tomography Enterograpy
ESGE	European Society of Gastrointestinal Endoscopy
HHT	Haemorrhagic Telangiectasia
FAP	Familiar Adenomatous Polyposis
FIT	Faecal Immunochemical Test
FPS	Frames Per Second
IBD	Inflammatory Bowel Disease
JPS	Juvenile Polyposis Syndrome
MAP	MUTYH-associated polyposis
MRE	Magnetic Resonance Enterography
NAP	NTHL-1 associated polyposis
PPAP	Polymerase Proofreading Associated Polyposis
PJS	Peutz-Jeghers Syndrome
PTEN-HS	Phosphatase and Tensin Homolog Hamartoma Syndrome
SBCE	Small Bowel Capsule Endoscopy
VCE	Video Capsule Endoscopy
